# Genetic association of *APOB* polymorphisms with variation in serum lipid profile among the Kuwait population

**DOI:** 10.1186/1476-511X-13-157

**Published:** 2014-10-08

**Authors:** Suzanne A Al-Bustan, Majed A Alnaqeeb, Babitha G Annice, Ghada A Ebrahim, Thanaa M Refai

**Affiliations:** Department of Biological Sciences, Faculty of Science, Kuwait University, PO Box 5969, SAFAT, 13060 Kuwait City, Kuwait; Yarmouk Clinic, Ministry of Health, Kuwait City, Kuwait; Department of Clinical Laboratories, Al-Amiri Hospital, Sharq, Kuwait City, Kuwait

**Keywords:** *APOB*, SNPs, VNTR, Genetic polymorphisms, Dyslipidemia, Genetic association, Arab, Kuwait

## Abstract

**Background:**

Several studies have identified *APOB* as a candidate gene predisposing individuals to dyslipidemia. Polymorphisms including the signal peptide (rs11279109), codon 2488 XbaI (rs1042031), codon 3611 MspI (rs693), codon 4154 EcoRI (rs1801701) and the 3’ variable number of tandem repeats have been reported to be associated with dyslipidemia in several populations. With limited studies on Arabs, this study aimed to investigate the genetic association of *APOB* polymorphisms and assess the potential influence of minor and rare alleles on serum lipid levels in the Kuwaiti population.

**Methods:**

A total of 795 Kuwaiti subjects, documented with phenotypic data and fasting serum lipid levels, were genotyped for the five polymorphisms using PCR, PCR-RFLP and gene fragment analysis. Genotype and allele association with variation in serum lipid levels as well as haplotypes were analyzed using chi-square test, univariate and logistic regression analysis.

**Results:**

Analysis of the genotype and allele frequencies distribution revealed a significant positive association between the *APOB* signal peptide and 3611 MspI polymorphisms with increased levels of triglycerides (statistical power of 80%). Haplotype analysis further supported the findings by showing that carriers of haplotypes (IX^−^M^−^E^+^M) had significantly lower mean (SD) TG levels (0.86 ± 0.07) as compared to non-carriers (1.01 ± 0.02). Significance was also observed with regards to positive family history of hypercholesterolemia.

**Conclusion:**

The results imply a “protective role” for two alleles (rs11279109 and rs1801701) in which logistic regression analysis showed a significant half–fold decrease in the risk for heterozygotes of rs11279109 and an 8.8 fold decrease in the risk for homozygous M^−^M^−^ of rs1801701 of having lower TG levels (<1.70 mmol/L) in individuals. This suggests that genetic interaction between various polymorphisms at different gene loci act in linkage disequilibrium to affect serum TG levels. Apo B genotyping may be a useful adjunct for the identification of individuals at risk of developing dyslipidemia in order to provide them with lifestyle modifications and/or pharmacological intervention to mitigate the effects of gene interaction and environmental influence.

**Electronic supplementary material:**

The online version of this article (doi:10.1186/1476-511X-13-157) contains supplementary material, which is available to authorized users.

## Background

The diet of Kuwaitis, majority of who are of Arab ethnicity, is relatively high in fat. The life style adopted by many Kuwaitis is thought to cause an increase in the levels of plasma lipids that may lead to an increase in risk factors leading to heart disease. The incidence of hypercholesterolemia was reported to be higher in women (36.6%) than in men (30.2%) while hypertriglyceridemia (HTG) was found to be higher in men (44.1%) than in women (33.8%) [1]. It has also been reported that the Kuwaiti population has one of the highest frequencies for overweight (80.4%), obesity (47.5%) and metabolic syndrome (36.2%) [2]. Several studies have attempted to elucidate the genetic and molecular mechanisms of dyslipidemia using measurements of plasma lipids and have identified a number of candidate genes and pathways relevant to lipid metabolism and/or transport [3–5]. In addition, several genome-wide association studies (GWAS) have established genetic association between variation in plasma lipid levels [6–8] with various gene loci being directly or indirectly involved in lipid metabolism and/or transport. In one GWAS study, a population case–control design that evaluated 1700 subjects at the extremes of lipid level phenotypes reported a significant association with the apolipoprotein B gene locus (*APOB)* as well as a significant accumulation of rare variants as defined by a minor allele frequency of <1% in controls [9].

The apoB protein forms the integral part of chylomicrons and VLDL synthesized during lipoprotein metabolism. The apoB-100 is a single polypeptide chain of 4,560-4,563-amino acids including a 27 residue signal sequence [10]. The mature protein contains the binding site for the uptake of LDL by the LDL receptor. The protein is coded by a 43 Kilo-base gene comprising 29 exons and 28 introns [11, 12]. There are several polymorphisms at the *APOB* gene locus [13–15]. These include a common polymorphism (rs11279109) in the promoter region coding for the signal peptide. The deletion allele (*sp24,* D*)* codes for the 24-amino acid signal peptide that lacks the hydrophobic leucine-alanine-leucine residues affecting protein hydrophobicity [16, 17], and has been shown to be associated with dyslipidemia, body mass index (BMI) and other phenotypes [18–22]. Other common polymorphisms are found in the coding sequences of exon 26, spanning 7572 bp [12] including a single nucleotide polymorphism (SNP) in the consensus sequence of the receptor-binding region [15] that can affect the binding affinity of the ligand to the LDL receptor [15, 23]. SNPs, including codons 3611 (rs693) and 4154 (rs1801701), result in amino acid substitution of glutamine to arginine and glutamic acid to lysine, respectively. These SNPs have been shown to be associated with variation in total cholesterol (TC) and low density lipoprotein-cholesterol (LDL-C) levels [20, 22, 24, 25]. In addition, another commonly studied polymorphism is found in the *APOB* gene in exon 29 (rs1042031) involves a silent mutation from cytosine to thymine in the third base of codon 2488 and has been reported to be associated with dyslipidemia [20, 22, 23, 25]. Finally, a variable number of tandem repeats (VNTR) at the 3’ end, located about 75 bp downstream the second polyadenylation signal [13] consisting of a dimeric 15 bp AT repeat unit [9, 14, 26], has been suggested to influence polyadenylation and processing of the mRNA transcript of apoB-100 [13].

The allele and genotype frequencies at the *APOB* gene locus have been studied and reported for numerous populations including Northern Europeans [27–29], Southern Europeans [19, 29, 30], Asians [22, 25, 31, 32] and others [33–36]. However, no such frequencies have ever been reported for the Kuwaiti population. Furthermore there are very few publications on the allele frequencies of the *APOB* polymorphisms in other Arab and Gulf region countries [21]. Generally, there has been very little data published on the genetic population structure of the Kuwaiti population [37–39]. Therefore the objective of this study was to investigate the genetic association of *APOB* polymorphisms with variation in lipid levels in the Kuwaiti population and to assess the potential influence of the minor and rare alleles on serum lipid levels. The Kuwaiti population is generally heterogeneous comprising two major ethnic groups, Arabs including Bedouin Arabs, and Persians, while the rest are an admix of both as well as other minor ethnic groups [38]. There are no previous studies on *APOB* polymorphisms in an Arab population at the *APOB* gene loci or with the association of the signal peptide, MspI and EcoRI polymorphisms with variation in serum lipid levels.

## Subjects and methods

### Sample description

Fasting blood samples were collected from a total of 795 healthy Kuwaiti volunteers whose informed consent for the purpose of the study was obtained. The study was reviewed and approved by the ethics committee at Kuwait University. The samples included 330 males and 465 females with ages ranging from 18 to 69 years of age and a mean of 57.1 years (±13.0 SD). The samples analyzed in this study included healthy Kuwaiti subjects undergoing routine medical checks at clinics or major hospitals in Kuwait. For each sample, phenotypic variables including BMI and family history of dyslipidemia, hypertension, diabetes and heart disease were recorded (Table 1).

**Table 1:** **General characteristics and serum lipid levels of the Kuwaiti population sample analyzed in this study**

	Male	Female	Total	P value
	(n = 330)	(n = 465)	(n = 795)	
Age (Years) ± SD	32.63 ± 0.78	30.62 ± 0.66	31.45 ± 0.68	0.0488
Body Mass Index (kg/m^2^) ± SD	27.24 ± 0.45	26.55 ± 0.35	26.80 ± 0.40	0.2264
Total Cholesterol (mmol/L) ± SD	4.71 ± 0.06	4.70 ± 0.05	4.70 ± 0.06	0.9037
Triglycerides (mmol/L) ± SD	1.28 ± 0.05	0.92 ± 0.04	1.06 ± 0.05	<0.0001
HDL-C (mmol/L) ± SD	0.97 ± 0.02	1.22 ± 0.02	1.13 ± 0.02	<0.0001
LDL-C (mmol/L) ± SD	3.19 ± 0.05	3.08 ± 0.04	3.12 ± 0.05	0.0837
**Ethnicity:**	**n (%)**	**n (%)**		
Arabs	126 (37.1)	214 (62.9)	340	0.1452
Bedouin Arabs	26 (44.1)	33 (55.9)	59	
Iranian	56 (42.4)	76 (57.6)	132	
Heterogeneous	122 (46.2)	142 (53.8)	264	
Positive Family History*:	**n (%)**	**n (%)**		
Dyslipidemia	86 (43.7)	111 (56.3)	197	
Hypertension	156 (39)	244 (61)	400	
Diabetes Mellitus 2	167 (38.7)	265 (61.3)	432	
Heart Disease	73 (36.0)	130 (64.0)	203	

*The numbers given is for those samples with documented family history only. Samples with undocumented history are not shown in any of the tables and were omitted from the statistical analysis and from the polymorphism association analysis.

### Biochemical analysis and diagnostic criteria

Quantitative measurements of serum TC, triglycerides (TG), high density lipoprotein-cholesterol (HDL-C), and LDL-C were determined by enzymatic methods with commercially available kits (Synchron System Reagents, Beckman Coulter, Brea, CA, USA) analyzed on a UniCel DxC 800 Synchron Clinical Systems (Beckman Coulter, USA) in the Clinical Chemistry Laboratory at Al-Amiri Hospital, Kuwait.

### Genotypic analysis of the *APOB*polymorphism

SNPs rs11279109, rs693, rs1801701, rs1042031, 3’VNTR were screened using various molecular techniques employing the polymerase chain reaction (PCR). The RFLP sites at the *APOB* gene locus include codon 2488 (rs693), codon 3611 (rs1801701) and codon 4154 (rs1042031) contain XbaI, MspI and EcoRI restriction sites, respectively. Total genomic DNA was extracted from 5 ml of whole blood using proteinase K digestion and salting out extraction according to the method described by Miller et al. [40]. The DNA samples were then subjected to genotyping for the five polymorphisms using different PCR-based techniques as summarized in Table 2.

**Table 2:** **Summary of the primers and PCR-RFLP conditions used for the analysis of the** ***APOB*** **polymorphisms**

SNP (RS#)	Primer sequence	Amplification conditions	PCR prod (bp)	RE digests	Ref
SP Ins/Del (rs11279109)	5’-CAGCTGGCGATGGACCCGCCGA-3’	95°C 5 mins	93/84	N/A	Xu et al. [41]
		95°C 0.50 sec, 65°C 1 min,			
	5’-ACCGGCCCTGGCGCCCGCCAGCA-3’	65°C 0.50 sec (35 cycles)			
		72°C 7 mins, hold at 4°C			
Codon 2488 Xba1 (rs693)	5’-GATGAAACCAATGACAAAATCC-3’	94°C 5 mins	450	240	Pan et al. [31]
	5’-AACAGTGAACCCTTGCTCTACC-3’	94°C 0.30 sec, 58°C 30 sec,			
		70°C 0.30 sec(40 cycles)		210	
		70°C 10 mins, hold at 4°C			
Codon 3611 Msp1 (rs1801701)	5’-AGAACATACAAGCAAAGCCA-3’	94°C 5 mins	273	169	Pan et al. [31]
	5’-GAGGAACCTTAGGTGTCCTTC-3’	94°C 0.30 sec, 56°C 30 sec,			
		70°C 0.30 sec(40 cycles)		104	
		70°C 10 mins, hold at 4°C			
Codon 4154 EcoR1 (rs1042031)	5’-TAGGCAAATTGATGATATCGA-3’	94°C 5 mins	330	181	Pan et al. [31]
	5’-ACCTGGGACAGTACCGTCCCTA-3’	94°C 0.30 sec, 54°C 30			
		sec,70°C 0.30 sec(40 cycles)			
				149	
		70°C 10 mins, hold at 4°C			
3’VNTR	5’-6- FAM- ATGGAAACGGAGAAATTATG-3’	95°C 5 mins	500-900	N/A	Alonso et al. [30]
		94°C 1 min, 58°C 1 min,			
	5’-CCTTCTCACTTGGCAAATAC-3’	72°C 4 mins (29 cycles)			
		72°C 7 mins, hold at 4°C			

#### Genotyping the signal peptide polymorphism at the APOB gene locus

The deletion/insertion allele at the signal peptide locus (rs11279109) was performed using PCR to amplify the target sequence (93 bp) as described by Xu et al*.* [41]. The PCR products were then analyzed by gel electrophoresis on a 4% 3:1 Nusieve: agarose gel pre-stained with ethidium bromide (10 mg/ml). Electrophoresis was performed for one hour at 200 V and 100 mA. Following electrophoresis, the bands were visualized under UV and documented using Syngene Digital Documentation system (Synoptics Ltd, Cambridge, UK). The fragment size was determined with Gene tools software (Version 4.00) by comparing the PCR product bands with those of a 123 bp DNA ladder.

#### *Genotyping the SNPS (*XbaI*,*MspI *and*EcoRI*) at the APOB Exon 26 and 29*

The genotypes for the three codons were determined by PCR-RFLP [24]. The PCR products (10 μl) were digested with the XbaI, MspI and EcoRI restriction enzymes in separate reactions [23]. The products were then separated by horizontal gel electrophoresis on a 4% 3:1 Nusieve:Agarose gel pre-stained with ethidium bromide (10 mg/ml). Following electrophoresis, the bands were visualized, documented and sized as described above. In order to ensure quality and reliability of the data obtained, randomly selected samples including all three genotypes at each locus (n = 10 for each SNP) were randomly selected for sequencing the target region to confirm the genotypes obtained by PCR-RFLP. The region spans the SNPs at XbaI, MspI and EcoRI of the *APOB* gene locus. These samples were then used as positive controls in subsequent PCR-RFLP reactions. All sequences were submitted to GenBank with the following accession numbers KC818417 (Xba1-Exon 26), KC818418 (Msp1-Exon 26) and KC818416 (Ecor1-Exon 29), respectively.

#### Genotyping and allele designation of the APOB 3’VNTR polymorphism

Amplification of the variable tandem repeats at the 3’ end flanking the second polyadenylation signal was carried out by PCR based on the method described by Alonso et al. [30] followed by gene fragment analysis on the ABI Gene Analyzer 3130xl (Applied Biosystems, Life Technologies, Paisly, UK). Allele designation of the 3’ VNTR locus at the *APOB* gene locus was established by determining the molecular size of the PCR products of the samples as compared with the ROX standard and positive controls. The positive controls were obtained by sequencing randomly selected samples (n = 10) to determine their exact size as base pairs and their corresponding alleles were designated according to Ludwig et al. [14]. To detect possible associations and simplify allele interaction analysis, the alleles were reassigned into a diallelic system where alleles <39 were coded as medium (M) and those that were ≥39 were coded as long (L).

### Analysis of genotype and allele frequency and Hardy-Weinberg equilibrium

The allele and genotype frequencies were determined by simple counting of all five polymorphisms including the signal peptide (rs11279109), XbaI (rs693), MspI (rs1801701), EcoRI (rs1042031) in the total population (n = 795) and with regards to plasma lipid levels and documented family history of various complex diseases (Table 1). The Hardy-Weinberg equilibrium (HWE) for the general population was estimated based on Chi-square using GENEPOP (Version 4.0.10) [42]. A p-value ≤ 0.05 indicated deviation from HWE.

### Haplotype construction and analysis

Linkage disequilibrium between the RFLP marker alleles at the *APOB* gene loci and their mutations have been reported to possibly have an effect on the phenotype, and that this may differ from one population to another [27]. Such SNPs can be used to construct haplotypes. In the present study, the 5 SNPs studied were used to construct the common haplotypes among the Kuwaiti population and their frequencies were estimated using Haploview 9 (Version 4.1). A total of 32 different haplotypes were recorded. Since the gametic phase of the samples is unknown, each sample was labeled as a carrier of the given haplotype based on their allelic combination for the five SNPs. In addition, univariate (ANOVA) analysis was carried out to investigate the association of the major haplotypes (>3%) observed with variation to lipid levels and other variables.

### Statistical analysis

Statistical analyses on the differences in the genotypic and allele frequency distribution were determined with regards to gender, BMI, and positive family history of hypercholesterolemia (HC), hypertriglyceridemia (HTG), hypertension (HT), diabetes mellitus (DM) and heart disease (HD) using the IBM SPSS Windows version 19.0. TG, TC, HDL-C, LDL-C values were natural log-transformed to achieve approximate normal distributions before further analysis. Chi-square tests were used to find the association between categorical variables including categorical lipid levels (lipid levels were divided into two main groups: normal levels and abnormal levels based on the cut-off value criteria of the American Heart Association for TC, TG, LDL-C and HDL-C). Moreover, genotype and allele frequency distribution for each SNP was compared with regards to the mean and standard deviation of plasma lipid levels using univariate ANOVA test. A two-tailed p-value of 0.05 was considered statistically significant. Bonferroni correction for multiple testing was taken into consideration but was not applied. In addition, logistic regression analyses were performed to investigate the association of the studied SNPs with lipid levels (Outcome variables: lipid levels, 0 = desirable levels and 1 = abnormal levels). Power calculations was estimated on a sample size of 100 from each ethnic group to detect the differences (delta) in the mean distribution of each polymorphism mean using the G*Power 3 statistical power analysis program [43]. Moreover, the sample size of n = 665 was analyzed using the Power and Sample Calculation Program (version 3.0.43) to detect power for the association of the significant SNPs with variation in lipid levels.

## Results

All 795 samples were successfully amplified and the expected PCR products of 93 bp for the insertion allele and the 84 bp for the deletion alleles were observed among all the samples (Additional file 1: Figure A). Table 3 summarizes the genotype and allele frequencies observed as well as the heterozygosity estimates for the signal peptide (rs11279109), XbaI (rs693), MspI (rs1801701) and EcoRI (rs1042031) polymorphisms.

**Table 3:** **Genotype and allele frequencies of the five** ***APOB*** **polymorphisms analyzed (n = 795)**

Polymorphism	Genotype			Allele		HWE	Heterozygosity estimate
**SP** (rs11279109)	**II**	**ID**	**DD**	**I**	**D**	p = 0.638	0.33
n	483	265	47	0.77	0.23		
(%)/f	60.75	33.33	5.91				
**VNTR**	**MM**	**ML**	**LL**	**M**	**L**	**p = 0.013**	0.15
n	655	116	24	0.90	0.10		
(%)/f	82.39	14.59	3.02				
**Xba1** (rs693)	**X** ^**−**^ **X** ^**−**^	**X** ^**+**^ **X** ^**−**^	**X** ^**+**^ **X** ^**+**^	**X** ^**−**^	**X** ^**+**^	p = 0.717	0.39
n	430	308	57	0.73	0.27		
(%)/f	54.09	38.74	7.17				
**Msp1** (rs1801701)	**M** ^**+**^ **M** ^**+**^	**M** ^**+**^ **M** ^**−**^	**M** ^**−**^ **M** ^**−**^	**M** ^**+**^	**M** ^**−**^	p = 0.599	0.12
n	692	99	4	0.93	0.07		
(%)/f	87.04	12.45	0.50				
**EcoR1** (rs1042031)	**E** ^**+**^ **E** ^**+**^	**E** ^**+**^ **E** ^**−**^	**E** ^**−**^ **E** ^**−**^	**E** ^**+**^	**E** ^**−**^	p = 0.211	0.18
n	640	142	13	0.89	0.11		
(%)/f	80.50	17.86	1.64				

The table summarizes the genotypes for the signal peptide (SP) (rs11279109), variable number of tandem repeats (3’VNTR), Codons 2488-XbaI C(X^−^) < T(X^+^) (rs693), 3611-MspI G(M^−^) < A(M^+^) (rs1801701) and 4154-EcoRI G(E^−^) < A(E^+^) (rs1042031).

n: number of samples, f: allele frequency HWE: Hardy-Weinberg Equilibrium with significant p- value level at 0.05.

Table 4 summarizes the results of logistic regression analysis with regards to TG levels only. The other lipid levels did not show any significance (Additional file 2: Tables A-C). The genotype and allele frequencies distribution for the major variables analyzed including variation to lipid levels and positive family history of dyslipidemia are summarized in Table 5. The results for all the variables analyzed with regards to all five *APOB* polymorphisms including univariate analysis are summarized in the Additional file 3: Tables A-E.

**Table 4:** **Results of logistic regression analysis of the** ***APOB*** **polymorphisms and TG levels (Outcome variable TG; 0 = ≤1.7 mmol/L and 1= >1.7 mmol/L)**

	Odds ratio (crude)	95% CI ^a^	p-value	Odds ratio (adjusted) ^b^	95% CI ^a^	p-value
SP (rs11279109)						
II	1.00			1.00		
ID	0.49	0.29 – 0.82	**0.007**	0.53	0.31 – 0.92	**0.023**
DD	1.04	0.44 – 2.45	0.931	1.01	0.41 – 2.48	0.990
Xba1 (rs693)						
X^−^X^−^	1.00			1.00		
X^+^X^−^	0.93	0.59 – 1.46	0.753	0.98	0.60 – 1.57	0.917
X^+^X^+^	0.76	0.31 – 1.88	0.558	0.82	0.32 – 2.11	0.680
Msp1 (rs1801701)						
M^+^M^+^	1.00			1.00		
M^+^M^−^	0.62	0.29 – 1.33	0.220	0.72	0.32 – 1.61	0.424
M^−^M^−^	5.81	0.81 – 41.82	0.080	8.80	1.13 – 68.46	**0.038**
EcoR1 (rs1042031)						
E^+^E^+^	1.00			1.00		
E^+^E^−^	0.95	0.53 – 1.70	0.867	0.93	0.50 – 1.71	0.810
E^−^E^−^	2.28	0.59 – 8.77	0.232	2.47	0.56 – 10.81	0.231
VNTR						
MM	1.00			1.00		
ML	0.65	0.32 – 1.29	0.217	0.66	0.32 – 1.37	0.264
LL	1.00	0.29 – 3.50	0.996	0.90	0.23 – 3.49	0.875

The table summarizes the results for the signal peptide (SP) (rs11279109), variable number of tandem repeats (3’VNTR), Codons 2488-XbaI C(X^−^) < T(X^+^) (rs693), 3611-MspI G(M^−^) < A(M^+^) (rs1801701) and 4154-EcoRI G(E^−^) < A(E^+^) (rs1042031).

^a^95% CI = 95% confidence interval for odds ratio with significant p- value level at 0.05.

^b^Adjusted for gender and age.

**Table 5:** **Distribution of genotype and allele frequencies of the** ***APOB*** **polymorphisms with the major variables analyzed among the Kuwaiti population (n = 795)**

	TC		TG		HDL-C		LDL-C		FH-HC		FH-HTG	
	Normal (≤5.17)	High (>5.17)	Normal (≤1.70)	High (>1.70)	Normal (>1.1 men & >1.29 women)	Abnormal (≤1.1 men & ≤1.29 women)	Normal (≤3.2)	High (>3.2)	Absent	Present	Absent	Present
**n**	475	191	571	95	211	418	389	238	409	197	594	12
***APOB*** **signal peptide (rs11279109)**												
**II n (%)**	277 (58.3)	118 (61.8)	338 (57.4)	67 (70.5)	118 (55.9)	258 (61.7)	233 (59.9)	141 (59.2)	248 (60.64)	121 (61.42)	364 (61.28)	5 (41.67)
**ID n (%)**	172 (36.2)	59 (30.9)	210 (36.8)	21 (22.1)	81 (38.4)	135 (32.3)	134 (34.5)	82 (34.5)	138 (33.7)	63 (32.0)	198 (33.3)	3 (25.0)
**DD n (%)**	26 (5.5)	14 (7.3)	33 (5.8)	7 (7.4)	12 (5.7)	25 (6.0)	22 (5.7)	15 (6.3)	23 (5.6)	13 (6.6)	32 (5.4)	4 (33.3)
**p**	0.338		**0.021**		0.313		0.944		0.839		**0.004**	
**D (f)**	0.236	0.228	0.242	0.184	0.249	0.221	0.229	0.235	0.225	0.226	0.221	0.458
**I (f)**	0.764	0.772	0.758	0.816	0.751	0.779	0.771	0.765	0.775	0.774	0.779	0.542
**p**	0.754		0.083		0.274		0.791		0.970		**0.006**	
***APOB*** **Codon 2488C (rs693)**												
**X** ^**−**^ **X** ^**−**^ **n (%)**	239 (50.3)	104 (54.5)	292 (51.1)	51 (53.7)	111 (52.6)	217 (52.6)	206 (53.0)	122 (51.3)	226 (55.3)	106 (53.8)	326 (54.9)	6 (50.0)
**X + X- n (%)**	199 (41.9)	73 (38.2)	234 (41.0)	38 (40.0)	84 (39.8)	168 (40.2)	152 (39.1)	98 (41.2)	153 (37.4)	79 (40.1)	229 (38.6)	3 (25.0)
**X** ^**+**^ **X** ^**+**^ **n (%)**	37 (7.8)	14 (7.3)	45 (7.9)	6 (6.3)	16 (7.6)	33 (7.9)	31 (8.0)	18 (7.6)	30 (7.3)	12 (6.1)	39 (6.6)	3 (25.0)
**p**	0.625		0.826		0.982		0.871		0.741		0.041	
**X** ^**−**^ **(f)**	0.713	0.736	0.716	0.737	0.725	0.720	0.725	0.718	0.740	0.739	0.742	0.625
**X** ^**+**^ **(f)**	0.287	0.264	0.284	0.263	0.275	0.280	0.275	0.282	0.260	0.261	0.258	0.375
**p**	0.399		0.559		0.851		0.805		0.969		0.198	
***APOB*** **Codon 3611–MspI G (rs1801701)**												
**M** ^**+**^ **M** ^**+**^ **n (%)**	407(85.7)	172(90.1)	494(86.5)	85(89.5)	180(85.3)	368(88.0)	337(86.6)	209(87.8)	362(88.5)	162(82.2)	516(86.9)	8(66.7)
**M** ^**+**^ **M** ^**−**^ **n (%)**	65(13.7)	18(9.4)	75(13.1)	8(8.4)	30(14.2)	47(11.2)	49(12.6)	28(11.8)	45(11.0)	35(17.8)	76(12.8)	4(33.3)
**M** ^**−**^ **M** ^**−**^ **n (%)**	3(0.6)	1(0.5)	2(0.4)	2(2.1)	1(0.5)	3(0.7)	3(0.8)	1(0.4)	2(0.5)	0(0.0)	2(0.3)	0(0.0)
**p**	0.315		**0.058**		0.532		0.822		**0.046**		0.113	
**M** ^**−**^ **(f)**	0.075	0.052	0.069	0.063	0.076	0.063	0.071	0.063	0.060	0.089	0.067	0.167
**M** ^**+**^ **(f)**	0.925	0.948	0.931	0.937	0.924	0.937	0.929	0.937	0.940	0.911	0.933	0.833
**p**	0.143		0.761		0.407		0.600		0.063		0.079	
***APOB*** **Codon 4154G (rs1042031)**												
**E** ^**+**^ **E** ^**+**^ **n (%)**	383(80.6)	154(80.6)	461(80.7)	76(80.0)	174(82.5)	334(79.9)	319(82.0)	187(78.6)	321(78.5)	158(80.2)	468(78.8)	11(91.7)
**E** ^**+**^ **E** ^**−**^ **n (%)**	83(17.5)	35(18.3)	102(17.9)	16(16.8)	34(16.1)	77(18.4)	63(16.2)	48(20.2)	82(20.0)	33(16.8)	114(19.2)	1(8.3)
**E** ^**−**^ **E** ^**−**^ **n (%)**	8(1.9)	2(1.1)	8(1.4)	3(3.2)	3(1.4)	7(1.7)	7(1.8)	3(1.3)	6(1.5)	6(3.0)	12(2.0)	0(0.0)
**p**	0.723		0.455		0.743		0.406		0.289		0.543	
**E** ^**−**^ **(f)**	0.106	0.102	0.103	0.116	0.095	0.109	0.099	0.113	0.115	0.114	0.116	0.042
**E** ^**+**^ **(f)**	0.894	0.898	0.897	0.884	0.905	0.891	0.901	0.887	0.885	0.886	0.884	0.958
**p**	0.820		0.604		0.441		0.416		0.971		0.257	
***APOB*** **3 VNTR**												
**MM n (%)**	391(82.3)	157(82.2)	466(81.6)	82(86.3)	174(82.5)	345(82.5)	321(82.5)	197(82.8)	339(82.9)	168(85.3)	496(83.5)	11(91.7)
**ML n (%)**	68(14.3)	30(15.7)	88(15.4)	10(10.5)	30(14.2)	62(14.8)	56(14.4)	35(14.7)	56(13.7)	22(11.2)	77(13.0)	1(8.3)
**LL n (%)**	16(3.4)	4(2.1)	17(3.0)	3(3.2)	7(3.3)	11(2.6)	12(3.1)	6(2.5)	14(3.4)	7(3.6	21(3.5)	0(0.0)
**p**	0.633		0.461		0.875		0.916		0.685		0.700	
**L (f)**	0.105	0.099	0.107	0.084	0.104	0.100	0.103	0.099	0.103	0.091	0.100	0.042
**M (f)**	0.895	0.901	0.893	0.916	0.896	0.900	0.897	0.901	0.897	0.909	0.900	0.958
**p**	0.754		0.343		0.834		0.816		0.537		0.503	

TC total cholesterol (expressed in mmol/L); TG triglycerides (expressed in mmol/L); LDL-C low density lipoprotein-cholesterol; HDL-C high density lipoprotein-cholesterol; FH-HTG positive family history of hypertriglyceridemia, FH-HC positive family history of hypercholesterolemia.

n = number of samples,% = frequency for each genotype in percent and ratio (f) for the allele.

The p-values were determined by chi-square test at a significance level of p = 0.05.

### Genotyping the signal peptide polymorphism at the *APOB*gene locus and its association to variation in lipid levels

The most common genotype observed (Table 3) was that for the homozygote insertion allele (n = 483, 60.75%) followed by the heterozygote allele (n = 265, 33.33%). Both the genotype and allele frequencies (I = 0.77, D = 0.23) were found to be in HWE (p > 0.05) with a mean heterozygosity value of 0.33.

The analysis (Table 5) showed a significant association between the insertion allele (p = 0.04) and increased levels of TG (>1.70 mmol/L, n = 95). There was a significantly (p = 0.021) lower frequency (22.1%) of heterozygotes with increased TG levels than heterozygotes with normal TG levels (36.8%). Regression analysis further confirmed the association by showing a half–fold risk for heterozygotes (Table 4). These results were further supported by the univariate analysis of the mean values of the lipid levels which also revealed a significant association between heterozygotes and variation in TG level (Additional file 3: Table A). The analysis showed that heterozygotes had significantly (p = 0.044) lower mean TG levels (0.95 ± 0.0) than homozygotes for either the deletion or the insertion allele. Moreover, a significant association was observed (p = 0.004) with regards to family history of HTG but the sample size of those with positive family history is too small to allow definite conclusions.

### Genotyping the SNPS (codons 2488, 3611 and 4154) at the *APOB*Exon 26 and 29 and its association with variation in lipid levels

All 795 samples were successfully amplified and the expected PCR products for each of the three polymorphisms (2488, 3611 and 4154) were observed (Additional file 1: Figure B-D). The expected genotypes were observed (Table 3) with the highest frequency being that for the homozygous wild type allele for all three SNPs with no significant differences or deviation from HWE (p > 0.05) among all the samples analyzed (n = 795). The highest mean heterozygosity estimate (0.39) was observed for the *APOB* codon 2488 XbaI SNP. The rare allele absent of the MspI restriction site for the *APOB* codon 3611 was found to be less than 1% (Table 3) contributing to the low mean heterozygosity estimate of 0.12. Similarly, the rare allele (absence of the restriction site) for the *APOB* EcoRI was found to be approximately 1.1% in the studied population also contributing to the low mean heterozygosity estimate (0.18).

No significant differences were observed with regards to variation in lipid levels and the other variables analyzed (Additional file 3: Table B) with the *APOB* XbaI polymorphism. The distribution of the genotype and allele frequencies was found to be similar among the different groups analyzed for the major variables (Table 5).

Analysis of the genotype and allele distribution for the *APOB* 3611 MspI polymorphism for the major variables analyzed (Table 5) revealed a probable significant association (p = 0.058) with serum TG levels. The rare allele M^−^ was observed to be higher in individuals with normal TG levels (n = 571) as indicated by the higher frequency (13.10%) of heterozygous samples carrying the rare M^−^ allele as compared to the 8.4% of individuals with increased levels of TG level (n = 95) and whom were mostly homozygotes (n = 85, 89.5%) for the wild type M^+^ allele implicating the M^−^ as a “protective allele”. Univariate analysis of the mean and standard deviation of the lipid levels revealed a possible significant association of heterozygous M^+^M^−^ with TG levels and BMI (Additional file 3: Table C). The analysis showed that heterozygotes showed a trend (p = 0.07) for lower mean TG levels (0.88 ± 0.9) than homozygous for either allele. Logistic regression analysis (Table 4) also showed a significant (p = 0.038) 8.8 fold increased probability of having lower TG levels (<1.70 mmol/L) in individuals with the homozygous M^−^M^−^ genotype. Moreover, individuals with positive family history of hypercholesterolemia (n = 197) had a significantly different genotype distribution (p = 0.046) where there were more heterozygous samples present (17.8%) than those without a positive family history of hypercholesterolemia (n = 409) (Table 5).

The analysis of the genotype and allele distribution for the *APOB* 4154 EcoRI polymorphism (Table 5) did not reveal any significant correlation with any of the lipid levels. Univariate analysis also did not show any significance with regards to the genotype distribution against the mean and standard deviation of serum lipid levels and BMI (Additional file 3: Table D).

### Genotyping and allele designation of the *APOB*3’VNTR polymorphism and its association to variation in lipid levels

**Figure 1:**
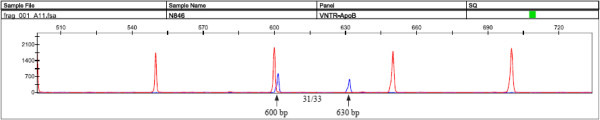
**An electropherogram of the** ***APOB*** **3’ VNTR polymorphism for a heterozygote sample generated by PCR and gene fragment analysis.** The peaks represent the alleles detected which are determined by comparison to a ROX standard. The sample shown was genotyped as 31/33 based on the number of repeats determined by their corresponding molecular size of 600 bp and 630 bp indicated with and an arrow on the figure. In the derived classification, this Kuwaiti sample was genotyped as M/L.

Gene fragment analysis of the 3’ VNTR locus (Figure 1) was found to be a simple and a rapid method for high-throughput genotyping. The data obtained proved to be highly variable in the Kuwaiti population with at least 43 different genotypes observed comprising 11 alleles ranging from as little as 31 repeats to as high as 51. In the derived diallelic classification, the most common genotype observed was that for the homozygous medium allele (n = 655, 82.39%) followed by the heterozygous (n = 116, 14.59%) and the least common was for the homozygous long allele (n = 24, 3.02%). The genotype frequencies for the derived classification of the M and L allele were found to deviate from the HWE (p = 0.013) contributing to the observed low mean heterozygosity value of 0.15 (Table 3). The deviation may also be the outcome of the cut-off values used in assigning the M and L alleles.

The genotype and allele frequencies were also compared among the different groups (Table 5) and with all the variables analyzed (Additional file 3: Table E). No significant findings were observed with regards to variation in lipid levels.

### Haplotype Analysis of the *APOB*gene polymorphisms and their associations to variations in lipid levels

All five polymorphisms were found to be in linkage disequilibrium (p ≤ 0.05) and hence were used to construct haplotypes. A total of 32 different haplotypes were observed among all the samples (n = 795). The haplotypes and their frequencies are summarized in Table 6. Haplotypes with less than a 3% frequency were grouped as other “O” haplotypes making up 24.1% of the total observed haplotypes. The most common observed haplotype “A” (28.9%) was that which consisted of all the wild type alleles at the five *APOB* loci while only one sample (0.04%) was observed with all rare alleles at the five loci. The haplotypes were examined for carrier status in all the samples and then were analyzed for their association with variation in lipid levels (Table 7). Carriers of haplotypes G (IX^−^M^−^E^+^M) had a significantly (p = 0.048) lower mean TG levels (0.86 ± 0.07) as compared to non-carriers whose mean TG levels were 1.01 ± 0.02.

**Table 6:** **Haplotypes at the** ***APOB*** **gene locus and their frequencies as observed in the population sample analyzed**

Haplotype	Alleles*	N	Frequency (%)
A	IX^−^M^+^R^+^M	681	28.9
B	IX^+^M^+^R^+^M	324	13.3
C	DX^−^M^+^R^+^M	262	11.1
D	DX^+^M^+^R^+^M	191	8.1
E	IX^−^M^+^R-M	137	5.8
F	IX^−^M^+^R^+^L	120	5.1
G	IX^−^M^−^R^+^M	73	3.1
O	-	568	24.1

The carrier status is defined as a sample which has all five alleles associated with the given haplotype and their frequency (%) is given. A sample may have more than one haplotype since the gametic phase is unknown. Haplotype “O” includes grouped haplotypes observed that were less than 3%.

*The alleles are for the polymorphisms in this order: rs11279109, rs693, rs1801701, rs1042031 and the 3’VNTR locus.

N = count number of carrier for each coded haplotype,**%** = frequency for each haplotype.

**Table 7:** **Association of the major haplotypes analyzed at the** ***APOB*** **gene locus and their association with variation in mean serum lipid levels (expressed in mmol/L) among the Kuwaiti samples analyzed**

Lipid	Haplotype	A	B	C	D	E	F	G	O
N	Non-carrier	1675	2032	2094	2165	2219	2236	2283	1788
	Carrier	681	324	262	191	137	120	73	568
TC	Non-carrier	4.67 ± 0.02	4.69 ± 0.02	4.69 ± 0.02	4.69 ± 0.02	4.69 ± 0.02	4.69 ± 0.02	4.69 ± 0.02	4.70 ± 0.02
	Carrier	4.72 ± 0.04	4.71 ± 0.05	4.70 ± 0.06	4.69 ± 0.07	4.73 ± 0.09	4.61 ± 0.10	4.68 ± 0.11	4.65 ± 0.04
	p value*	**0.356**	**0.767**	**0.879**	**0.937**	**0.667**	**0.417**	**0.935**	**0.298**
TG	Non-carrier	1.00 ± 0.02	1.07 ± 0.03	1.01 ± 0.02	1.01 ± 0.02	1.01 ± 0.02	1.01 ± 0.02	1.01 ± 0.02	1.03 ± 0.02
	Carrier	1.05 ± 0.05	1.05 ± 0.03	1.00 ± 0.05	0.97 ± 0.05	1.05 ± 0.08	1.02 ± 0.09	0.86 ± 0.07	0.95 ± 0.03
	p value*	**0.334**	**0.728**	**0.914**	**0.998**	**0.440**	**0.707**	**0.048**	**0.089**
HDL-C	Non-carrier	1.14 ± 0.01	1.12 ± 0.01	1.13 ± 0.01	1.13 ± 0.01	1.14 ± 0.01	1.14 ± 0.01	1.13 ± 0.01	1.14 ± 0.01
	Carrier	1.14 ± 0.02	1.14 ± 0.01	1.16 ± 0.02	1.16 ± 0.03	1.11 ± 0.03	1.10 ± 0.03	1.20 ± 0.04	1.13 ± 0.01
	p value*	**0.800**	**0.180**	**0.238**	**0.334**	**0.324**	**0.204**	**0.147**	**0.577**
LDL-C	Non-carrier	3.12 ± 0.02	3.13 ± 0.03	3.12 ± 0.02	3.12 ± 0.02	3.11 ± 0.02	3.11 ± 0.02	3.12 ± 0.02	3.12 ± 0.02
	Carrier	3.11 ± 0.05	3.11 ± 0.03	3.11 ± 0.05	3.10 ± 0.06	3.17 ± 0.07	3.12 ± 0.09	3.10 ± 0.11	3.10 ± 0.04
	p value*	**0.935**	**0.688**	**0.954**	**0.816**	**0.476**	**0.923**	**0.866**	**0.568**

The table shows the mean and standard deviation (SD) of serum lipid levels using univariate Anova (UNIANOVA). A two-tailed (t-test) p-value of 0.05 was considered as statistical significance. The carrier status is defined as a sample which has all five alleles associated with the given haplotype and their count (N) is given. A sample may have more than one haplotype since the gametic phase is unknown.

N count number; TC total cholesterol; TG triglycerides; LDL-C low density lipoprotein-cholesterol; HDL-C high density lipoprotein-cholesterol.

*t-test p-value of 0.05 was considered as statistical significance.

## Discussion

This study provides a comprehensive analysis of five *APOB* polymorphisms and their correlation with variations of serum lipid levels in the Kuwaiti population. Significant findings were observed for the genetic association between the *APOB* signal peptide (rs11279109) and MspI (rs1801701) polymorphisms with variation in TG levels among the Kuwaiti samples analyzed. Heterozygous samples at the *APOB* signal peptide locus were significantly (p = 0.023) associated with lower TG serum levels (Tables 4 and 5). This may suggest an interaction between the two alleles to influence serum TG levels and thus genetically predispose individuals to dyslipidemia. This may also explain why several studies investigating the role of this SNP with variation in lipid levels and other disorders reported conflicting results. The two alleles were found at frequencies of more than 5% with an average heterozygosity estimate (0.33) revealing that both alleles could possibly exhibit different influences on serum lipid levels in different ethnic groups. Furthermore, univariate analysis of the *APOB* signal peptide polymorphism also revealed a significant (p = 0.021) association between carriers of the insertion allele with lower mean serum TG. These abnormalities in lipid profile associated with the *APOB* signal peptide polymorphism may be the result of a change in the degree of hydrophobicity and efficacy of apoB processing [16, 41, 44] due to either the presence or absence of the hydrophobic amino acid residues [16, 41, 44]. This is also reflected in the results obtained from the logistic regression (Table 4) analysis (both adjusted and unadjusted) which showed a half fold decrease in the risk of having increased TG levels for carriers of the insertion allele.

The SNP at *APOB* codon 3611 involves the loss of the ancestral restriction sites of MspI [13] as a result of a transition mutation changing a G to an A at codon 3611 in exon 26. In this study, most of the individuals (95.92%) with increased TG levels were homozygous for the wild type M^+^ allele. Among the Kuwaiti population, the rare M^−^ allele (p = 0.031) was observed to have a “protective” role exhibiting an 8.8 fold decrease in the risk of high TG levels in individuals homozygous for the rare M^−^ allele. Some of the subjects in the present study with positive family history of hypercholesterolemia (n = 197) also showed a significant association (p = 0.046) with the rare M^−^ allele where there was a higher frequency of heterozygotes (17.77%) when compared to those samples with no family history of hypercholesterolemia (n = 409). Interestingly, none of these heterozygotes had increased levels of serum TG further supporting the “protective” role of the M^−^ allele. It has been documented that the missense substitution resulting from the loss of the MspI RE site may affect apoB-100 protein function; LDL-receptor recognition and binding affinity resulting in abnormal lipid metabolism and levels, and possibly body fat accumulation [23]. The extent of interaction of various mutations in different ethnic groups may also influence the levels, possibly explaining the reason behind conflicting results from different populations.

The inconsistent reports could also be attributed to linkage disequilibrium of the MspI polymorphism with other polymorphisms in genes responsible for lipid metabolism and transport as well as to the diverse biological functions affected by this polymorphism. Haplotype analysis of the five polymorphisms also implicated the same two SNPs indicating the potential effect of *APOB* on serum TG levels. It was observed that carriers for the *APOB* haplotype consisting of the IX^−^M^−^E^+^M (3.11%) had a significantly (p = 0.048) lower mean serum TG level further supporting the “protective” role for both the insertion allele at the signal peptide and the M^−^ at the MspI loci on serum TG levels. As observed for the MspI polymorphism in this study, Johansen et al. [9] reported that although there was a possibility of finding few HTG patients with very low risk allele scores as well as few normal individuals with very high risk allele scores, a considerable overlap for a central distribution of risk allele scores could also be found. Since the risk allele TG scores could discriminate between normal individuals and those with the risk of developing HTG based on the extreme values, such identification could aid early intervention.

The results from the present study reflect a genetic predisposition to dyslipidemia in the Kuwaiti population that is unlikely to be attributed to population stratification since there were no statistical differences (p > 0.05) observed between the four ethnic groups that make up the Kuwaiti population. The genotype and allelic distribution for the *APOB* polymorphisms in the Kuwaiti population were found to be somewhat different when compared to other populations (Table 8). The allelic frequencies for the signal peptide polymorphism was found to be most similar to that reported for another Arab population, the Tunisian population [21], and somewhat different from some Southeast Asian populations. There are no available frequencies for other Arab ethnic populations for the MspI polymorphism so the frequencies in this study resembled somewhat those reported for Caucasians [45] and those for the Turkish population [36]. The similar frequencies found in the Kuwaiti population, the Tunisians and Turks could be attributed to their common Arab ancestry.

**Table 8:** **Comparison of the allelic frequencies reported in this study and other studies at the** ***APOB*** **gene locus**

Population	n	Ins/Del		Xba1		Msp1		EcoR1		Reference
		I	D	X ^−^	X ^+^	M ^+^	M ^−^	R ^+^	R ^−^	
Tunisian	458	0.743	0.257	0.725	0.275					Kallel et al. [21]
Caucasians	111			0.470	0.530	0.940	0.060	0.790	0.210	Genest et al. [45]
Indian	181	0.892	0.108	0.830	0.170			0.900	0.100	Saha et al. [46]
	100			0.775	0.225	0.850	0.150	0.820	0.180	Puri et al. [32]
Srilankan	190					0.981	0.019	0.969	0.031	Mendis et al. [47]
Turkish	250			0.690	0.310			0.830	0.170	Duman et al. [36]
Danish	464			0.51	0.49	0.913	0.087	0.83	0.17	Hansen et al. [27]
Greek	90			0.500	0.500	0.899	0.101	0.819	0.181	De Benedictis et al. [48]
Mongolians	110	0.909	0.091	0.936	0.064	0.991	0.009	0.950	0.050	Tsunoda et al. [22]
Chinese	288	0.793	0.207	0.909	0.091	0.928	0.072	0.929	0.071	Saha et al. [49]
Japanese	1328	0.831	0.169	0.960	0.040			0.934	0.066	Zaman et al. [50]
Bulgarians	147	0.793	0.207	0.568	0.432	0.959	0.041	0.816	0.184	Horvath et al. [19]
Nigerians	1222	0.749	0.251	0.843	0.158					Anderson et al. [33]
Kuwait General Population	795	0.774	0.226	0.735	0.265	0.933	0.067	0.894	0.106	Present Study
Kuwait HTG	49	0.847	0.153	0.745	0.255	0.969	0.031	0.949	0.051	Present Study
Kuwait DYS	238	0.746	0.254	0.721	0.279	0.922	0.078	0.895	0.105	Present Study

HTG hypertriglyceridemia; DYS dyslipidemia marked by abnormal LDL-C levels.

The results from our study agree with studies that correlate genetics, ethnicity and environmental factors with dyslipidemia [21, 22]. The combination of these factors could explain the inter-individual variations in lipid levels. Power calculations of 69% at a 5% significance for the subpopulation sample size analyzed [43] and a power of 80% at a 5% significance in the difference of the mean level of TG in addition to the positive association of the *APOB* insertion/deletion and MspI polymorphisms with variation in serum TG levels suggests a strong genetic predisposition to dyslipidemia for the Kuwaiti population. Unlike the present study most reports have indicated an association with LDL-C, and only recently did GWAS population-based studies report an association with TG levels implicating *APOB* S4338I polymorphism (rs4635554) with increased risk to HTG [9]. Although the polymorphism detected by GWAS is different from those analyzed in this study, it still implies genetic influence of *APOB* on variation in serum lipid levels. Johansen et al. [9] suggested that it is possible that the accumulation of “rare” alleles at different loci, identified and those unidentified may cause TG level variations and dyslipidemia by an unknown molecular mechanism.

## Conclusion

Studies investigating the possible association of various *APOB* polymorphisms with specific metabolic disorders or syndromes reported inconsistent findings as well as some conflicting conclusions. The results presented here mainly showed an association of *APOB* polymorphisms with variation in serum TG levels among the Kuwaiti population which has not been reported previously. The results may reflect failings in the underlying mechanisms of lipid transport and metabolism, hence leading to abnormal plasma lipid levels. The results supports the need to identify individuals who are at risk of developing HTG and/or dyslipidemia in order to provide them with lifestyle modifications such as personalized nutrition and/or physical activity. Since lipid levels may be genetically controlled, the identification and characterization of genetic variants associated with plasma lipid concentrations can provide useful information related to genotype-phenotype relationships [9] and the role of ethnicity. The agreement of this study with others reporting positive association of various *APOB* polymorphisms with variation in TG levels and dyslipidemia, as well as the inconsistent frequencies reported for different populations, strongly supports the need for the complete sequencing of the *APOB* gene in order to identify known common functional variants and rare “risk” or “protective” alleles. In addition further elucidation of inter-individual and population variations that may affect serum TG and other lipid levels is important. One major limitation of this study was the lack of serum levels of apolipoproteins which could have provided further insight into the mechanisms of the significant SNPs identified leading to the effect on the apolipoprotein levels. Finally, lack of association with the 3 of the polymorphisms analyzed (rs693, rs1042031 and the 3’VNTR) could have been due to lack of statistical power as a result of missing data.

## Electronic supplementary material

Additional file 1: **Includes four figures illustrating the banding patterns for the PCR products of the** ***APOB*** **signal peptide polymorphism (Figure A) and PCR products digested with the XbaI for the codon 2488 polymorphism (Figure B), MspI for codon 3611 (Figure C) and EcoRI for codon 4154 (Figure D).** The figure legend explains the banding patterns expected and their corresponding genotypes. (PDF 211 KB)

Additional file 2: **Includes logistic regression analysis of all five** ***APOB*** **polymorphisms with TC, HDL and LDL levels.** The file includes a total of 3 tables. (DOC 136 KB)

Additional file 3: **Includes complete data analysis for all the variables analyzed in this study with regards to all five** ***APOB*** **polymorphisms employing chi-square test and univariate ANOVA.** The file includes a total of 10 tables, 2 tables for each polymorphism. The significance values are highlighted and were incorporated into the final manuscript. (DOCX 46 KB)

## Abbreviations

ANOVA: Analysis of variance
*APOB*: Apolipoprotein B gene
*APOB*-SP: Apolipoprotein B-signal peptide
BMI: Body mass index
DNA: Deoxyribonucleic acid
DM: Diabetes mellitus
EcoR1: E.coli restriction enzyme I
DNA: Deoxyribonucleic acid
GWAS: Genome wide association studies
FH-HC: Family history of hypercholesterolemia
FH-HTG: Family history of hypertriglyceridemia
HD: Heart disease
HDL-C: High-density-lipoprotein-cholesterol
HDL: High-density lipoprotein
HTG: Hypertriglyceridemia
HWE: Hardy-Weinberg equilibrium
LDL: Low-density lipoprotein
LDL-C: Low-density lipoprotein-cholesterol
MspI: Moraxella sp. type I restriction enzyme
mRNA: Messenger ribonucleic acid
PCR: Polymerase chain reaction
RE: Restriction enzymes
RFLP: Restriction fragment length polymorphism
SPSS: Statistical Package for the Social Sciences
SNPs: Single nucleotide polymorphisms
TC: Total cholesterol
TG: Triglycerides
VLDL: Very low density lipoproteins
VNTR: Variable number tandem repeats
UNI ANOVA: Univariate analysis of variance
XbaI: Xanthomonas badrii type I restriction enzyme.
