# Supplementary material for: Genetic association of APOB polymorphisms with variation in serum lipid profile among the Kuwait population
Source: Lipids Health Dis. 2014 Oct 8;13:157. doi: 10.1186/1476-511X-13-157 (PMC4201729; doi:10.1186/1476-511X-13-157)
Supplement: Supplementary file 1 — Additional file 1: Includes four figures illustrating the banding patterns for the PCR products of the APOB signal peptide polymorphism (Figure A) and PCR products digested with the XbaI for the codon 2488 polymorphism (Figure B), MspI for codon 3611 (Figure C) and EcoRI for codon 4154 (Figure D). The figure legend explains the banding patterns expected and their corresponding genotypes. (PDF 211 KB) [file 12944_2014_1139_MOESM1_ESM.pdf]

Figure A-D . PCR and PCR-RFLP products (Lanes 1-8), their molecular size (bp) and their corresponding genotypes of the APOB gene locus resolved on a 3.5% 3:1 Nusieve:Agarose gel stained with 10mg/ml ethidium bromide. Genotyping of APOB polymorphisms was achieved by determining the size of the products resolved on the gel of each allele when compared to a 50bp DNA ladder (M). A negative (N) and a positive control (lanes 1 & 9).

(A) PCR products of the APOB signal peptide insertion (I)/deletion (D) (rs11279109) polymorphism. The expected products are 94bp for a homozygous II (Lane 4), 84bp for a homozygous DD (Lane 5) and both for a heterozygous ID (Lane 2).

(B) PCR-RFLP products of the APOB Codon 2488C(X^-)<T(X^+) (rs693) polymorphism. The expected products are 450bp for a homozygous X^-X^- (Lane 2), 240bp and 210 for a homozygous X^+X^+ (Lane 5) and all three for a heterozygous X^+X^- (Lane 4).

(C) PCR-RFLP products of the APOB Codon 3611-MspI G(M^-)<A(M^+) (rs1801701) polymorphism. The expected products are 273bp for a homozygous M^-M^- (not observed), 169bp and 104 for a homozygous M^+M^+ (Lane 2) and all three for a heterozygous M^+M^- (Lane 7).

(D) PCR-RFLP products the APOB Codon 4154G(E^+)<A(E^-) (rs1042031) polymorphism. The expected products are 330bp for a homozygous E^-E^- (not observed), 181bp and 149 for a homozygous E^+E^+(Lane 7) and all three for a heterozygous E^+E^- (Lane 8).

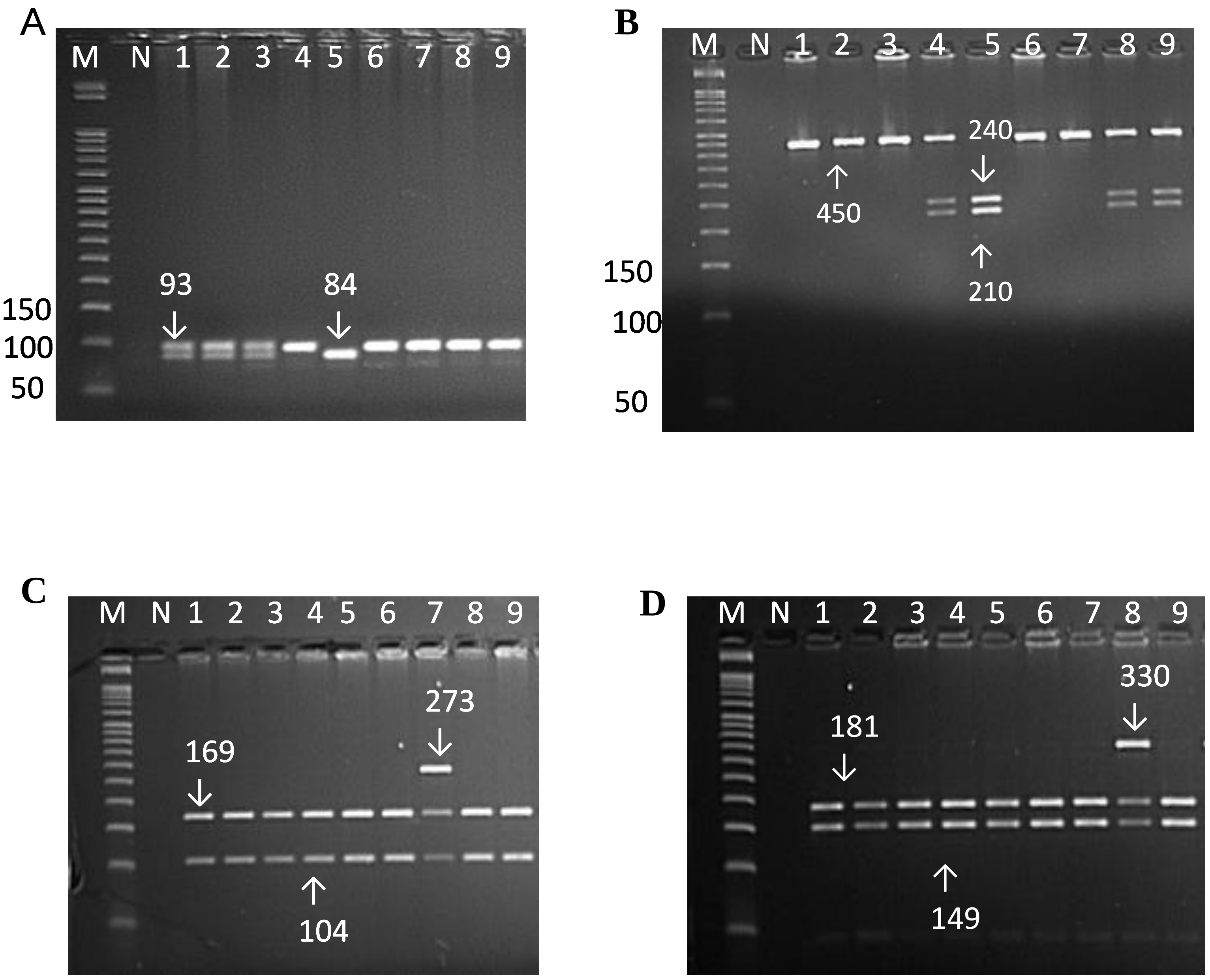
A
M N 1 2 3 4 5 6 7 8 9
93
84
150
100
50
B
M N 1 2 3 4 5 6 7 8 9
240
450
210
150
100
50
C
M N 1 2 3 4 5 6 7 8 9
273
169
104
D
M N 1 2 3 4 5 6 7 8 9
330
181
149
