# Supplementary material for: Genetic association of APOB polymorphisms with variation in serum lipid profile among the Kuwait population
Source: Lipids Health Dis. 2014 Oct 8;13:157. doi: 10.1186/1476-511X-13-157 (PMC4201729; doi:10.1186/1476-511X-13-157)
Supplement: Supplementary file 2 — Additional file 2: Includes logistic regression analysis of all five APOB polymorphisms with TC, HDL and LDL levels. The file includes a total of 3 tables. (DOC 136 KB) [file 12944_2014_1139_MOESM2_ESM.doc]

Table A. Results of logistic regression analysis of the APOB polymorphisms and TC levels (Outcome variable TC; 0= ≤5.17mmol/L and 1= >5.17mmol/L). The table summarizes the results for the signal peptide (SP) (rs11279109), variable number of tandem repeats (VNTR), Codons 2488-XbaI C(X^-^) <T(X^+^) (rs693), 3611-MspI G(M^-^)<A(M^+^) (rs1801701) and 4154-EcoRI G(R^-^)<A(R^+^) (rs1042031).

|  |  |  |  |  |  |  |  |
| --- | --- | --- | --- | --- | --- | --- | --- |
|  | Odds ratio |  |  |  | Odds ratio |  |  |
|  | (crude) | 95% CI^a^ | p-value |  | (adjusted)^b^ | 95% CI^a^ | p-value |
|  |  |  |  |  |  |  |  |
|  |  |  |  |  |  |  |  |
| SP |  |  |  |  |  |  |  |
| II | 1.00 |  |  |  | 1.00 |  |  |
| ID | 0.81 | 0.56 – 1.16 | 0.246 |  | 0.85 | 0.58 – 1.24 | 0.398 |
| DD | 1.26 | 0.64 – 2.51 | 0.502 |  | 1.28 | 0.63 – 2.59 | 0.492 |
| Xba1 |  |  |  |  |  |  |  |
| X^-^X^-^ | 1.00 |  |  |  | 1.00 |  |  |
| X^+^X^-^ | 0.84 | 0.59 – 1.20 | 0.344 |  | 0.86 | 0.60 – 1.24 | 0.416 |
| X^+^X^+^ | 0.87 | 0.45 – 1.68 | 0.677 |  | 0.90 | 0.46 – 1.76 | 0.754 |
| Msp1 |  |  |  |  |  |  |  |
| M^+^M^+^ | 1.00 |  |  |  | 1.00 |  |  |
| M^+^M^-^ | 0.66 | 0.38 – 1.14 | 0.133 |  | 0.74 | 0.42 – 1.30 | 0.292 |
| M^-^M^-^ | 0.79 | 0.08 – 7.64 | 0.838 |  | 0.83 | 0.08 – 8.17 | 0.871 |
| EcoR1 |  |  |  |  |  |  |  |
| R^+^R^+^ | 1.00 |  |  |  | 1.00 |  |  |
| R^+^R^-^ | 1.05 | 0.68 – 1.62 | 0.831 |  | 1.02 | 0.65 – 1.60 | 0.929 |
| R^-^R^-^ | 0.55 | 0.12 – 2.59 | 0.451 |  | 0.53 | 0.11 – 2.60 | 0.437 |
| VNTR |  |  |  |  |  |  |  |
| MM | 1.00 |  |  |  | 1.00 |  |  |
| ML | 1.10 | 0.69 – 1.75 | 0.693 |  | 1.17 | 0.72 – 1.89 | 0.524 |
| LL | 0.62 | 0.21 – 1.89 | 0.403 |  | 0.57 | 0.18 – 1.80 | 0.338 |
|  |  |  |  |  |  |  |  |

^a^95% CI = 95% confidence interval for odds ratio. ^b^Adjusted by gender and age.

Table B. Results of logistic regression analysis of the APOB polymorphisms and HDL levels (Outcome variable HDL ratio; 0= >1.1 for men & >1.29 for women and 1= <=1.1 for men & <=1.29 for women). The table summarizes the results for the signal peptide (SP) (rs11279109), variable number of tandem repeats (VNTR), Codons 2488-XbaI C(X^-^)<T(X^+^) (rs693), 3611-MspI G(M^-^) <A(M^+^) (rs1801701) and 4154-EcoRI G(R^-^)<A(R^+^) (rs1042031).

|  |  |  |  |  |  |  |  |
| --- | --- | --- | --- | --- | --- | --- | --- |
|  | Odds ratio |  |  |  | Odds ratio |  |  |
|  | (crude) | 95% CI^a^ | p-value |  | (adjusted)^b^ | 95% CI^a^ | p-value |
|  |  |  |  |  |  |  |  |
|  |  |  |  |  |  |  |  |
| SP |  |  |  |  |  |  |  |
| II | 1.00 |  |  |  | 1.00 |  |  |
| ID | 0.76 | 0.54 – 1.08 | 0.130 |  | 0.80 | 0.56 – 1.15 | 0.224 |
| DD | 0.95 | 0.46 – 1.96 | 0.896 |  | 0.91 | 0.44 – 1.90 | 0.799 |
| Xba1 |  |  |  |  |  |  |  |
| X^-^X^-^ | 1.00 |  |  |  | 1.00 |  |  |
| X^+^X^-^ | 1.02 | 0.72 – 1.45 | 0.898 |  | 1.02 | 0.72 – 1.46 | 0.908 |
| X^+^X^+^ | 1.06 | 0.56 – 2.00 | 0.870 |  | 1.08 | 0.56 – 2.06 | 0.823 |
| Msp1 |  |  |  |  |  |  |  |
| M^+^M^+^ | 1.00 |  |  |  | 1.00 |  |  |
| M^+^M^-^ | 0.77 | 0.47 – 1.25 | 0.288 |  | 0.82 | 0.50 – 1.37 | 0.454 |
| M^-^M^-^ | 1.47 | 0.15 – 14.21 | 0.741 |  | 1.64 | 0.17 – 16.19 | 0.672 |
| EcoR1 |  |  |  |  |  |  |  |
| R^+^R^+^ | 1.00 |  |  |  | 1.00 |  |  |
| R^+^R^-^ | 1.18 | 0.76 – 1.84 | 0.465 |  | 1.19 | 0.76 – 1.86 | 0.461 |
| R^-^R^-^ | 1.22 | 0.31 – 4.76 | 0.779 |  | 2.21 | 0.30 – 4.86 | 0.788 |
| VNTR |  |  |  |  |  |  |  |
| MM | 1.00 |  |  |  | 1.00 |  |  |
| ML | 1.04 | 0.65 – 1.67 | 0.863 |  | 1.05 | 0.65 – 1.70 | 0.842 |
| LL | 0.79 | 0.30 – 2.08 | 0.637 |  | 0.79 | 0.30 – 2.13 | 0.648 |
|  |  |  |  |  |  |  |  |

^a^95% CI = 95% confidence interval for odds ratio. ^b^Adjusted by gender and age.

Table C. Results of logistic regression analysis of the APOB polymorphisms and LDL levels (Outcome variable LDL ratio; 0= ≤3.2 and 1= >3.2). The table summarizes the results for the signal peptide (SP) (rs11279109), variable number of tandem repeats (VNTR), Codons 2488-XbaI C(X^-^) <T(X^+^) (rs693), 3611-MspI G(M^-^) <A(M^+^) (rs1801701) and 4154-EcoRI G(R^-^)<A(R^+^) (rs1042031).

|  |  |  |  |  |  |  |  |
| --- | --- | --- | --- | --- | --- | --- | --- |
|  | Odds ratio |  |  |  | Odds ratio |  |  |
|  | (crude) | 95% CI^a^ | p-value |  | (adjusted)^b^ | 95% CI^a^ | p-value |
|  |  |  |  |  |  |  |  |
|  |  |  |  |  |  |  |  |
| SP |  |  |  |  |  |  |  |
| II | 1.00 |  |  |  | 1.00 |  |  |
| ID | 1.01 | 0.72 – 1.43 | 0.950 |  | 1.06 | 0.75 – 1.51 | 0.730 |
| DD | 1.13 | 0.57 – 2.44 | 0.734 |  | 1.10 | 0.55 – 2.22 | 0.790 |
| Xba1 |  |  |  |  |  |  |  |
| X^-^X^-^ | 1.00 |  |  |  | 1.00 |  |  |
| X^+^X^-^ | 1.09 | 0.78 – 1.53 | 0.623 |  | 1.04 | 0.71 – 1.52 | 0.843 |
| X^+^X^+^ | 0.98 | 0.53 – 1.83 | 0.950 |  | 0.71 | 0.37 – 1.36 | 0.306 |
| Msp1 |  |  |  |  |  |  |  |
| M^+^M^+^ | 1.00 |  |  |  | 1.00 |  |  |
| M^+^M^-^ | 0.92 | 0.56 – 1.51 | 0.746 |  | 1.04 | 0.63 – 1.44 | 0.868 |
| M^-^M^-^ | 0.54 | 0.06 – 5.20 | 0.592 |  | 0.56 | 0.06 – 5.51 | 0.621 |
| EcoR1 |  |  |  |  |  |  |  |
| R^+^R^+^ | 1.00 |  |  |  | 1.00 |  |  |
| R^+^R^-^ | 1.30 | 0.86 – 1.97 | 0.218 |  | 1.28 | 0.84 – 1.96 | 0.254 |
| R^-^R^-^ | 0.73 | 0.19 – 2.86 | 0.653 |  | 0.70 | 0.17 – 2.82 | 0.613 |
| VNTR |  |  |  |  |  |  |  |
| MM | 1.00 |  |  |  | 1.00 |  |  |
| ML | 1.02 | 0.64 – 1.61 | 0.938 |  | 0.92 | 0.56 – 1.53 | 0.750 |
| LL | 0.82 | 0.30 – 2.21 | 0.687 |  | 0.65 | 0.24 – 1.77 | 0.394 |
|  |  |  |  |  |  |  |  |

^a^95% CI = 95% confidence interval for odds ratio. ^b^Adjusted by gender and age.
