# Supplementary material for: Genetic association of APOB polymorphisms with variation in serum lipid profile among the Kuwait population
Source: Lipids Health Dis. 2014 Oct 8;13:157. doi: 10.1186/1476-511X-13-157 (PMC4201729; doi:10.1186/1476-511X-13-157)
Supplement: Supplementary file 3 — Additional file 3: Includes complete data analysis for all the variables analyzed in this study with regards to all five APOB polymorphisms employing chi-square test and univariate ANOVA. The file includes a total of 10 tables, 2 tables for each polymorphism. The significance values are highlighted and were incorporated into the final manuscript. (DOCX 46 KB) [file 12944_2014_1139_MOESM3_ESM.docx]

**Table A: Distribution of the genotypic and allelic frequencies of the APOB signal peptide insertion (I)/deletion (D) polymorphism (rs11279109) with serum lipid levels in the Kuwaiti population (n=795).**

A.1 Frequency distribution against all the variables analyzed in the study. The serum lipids were categorized based on the Kuwaiti population reference values (mmol/L) as indicated in the tables.

| **Variable** | **N** | **GENOTYPE** | | | | **ALLELE** | | |
| --- | --- | --- | --- | --- | --- | --- | --- | --- |
|  |  | DD | ID | II | P value* | D | I | p-value* |
|  |  | N (%) | N (%) | N (%) |  | f | f |  |
| **Gender** Male  Female | 329  462 | 23 (7.0)  24 (5.2) | 104 (31.6)  159 (34.4) | 202 (61.4)  279 (60.4) | 0.49 | 0.228  0.224 | 0.772  0.776 | 0.85 |
| **TC** Normal (≤5.17)  High (>5.17) | 475  191 | 26 (5.5)  14 (7.3) | 172 (36.2)  59 (30.9) | 277 (58.3)  118 (61.8) | 0.34 | 0.236  0.228 | 0.764  0.772 | 0.75 |
| **TG** Normal (≤2.19)  High (>2.19) | 617  49 | 37 (6.0)  3 (6.1) | 222 (36.0)  9 (18.4) | 358 (58.0)  37 (75.5) | 0.03 | 0.240  0.153 | 0.760  0.847 | 0.04 |
| **HDL-C** Normal (>1.3)  Abnormal (≤1.3) | 276  353 | 15 (5.4)  22 (6.2) | 110 (39.9)  106 (30.0) | 151 (54.7)  225 (63.7) | 0.04 | 0.254  0.212 | 0.746  0.788 | 0.09 |
| **LDL-C** Normal (≤3.2)  High (>3.2) | 389  238 | 22 (5.7)  15 (6.3) | 134 (34.4)  82 (34.4) | 233 (59.9)  141 (59.2) | 0.94 | 0.229  0.235 | 0.771  0.765 | 0.79 |
| **FH-HT** Absent  Present | 207  400 | 9 (4.3)  27 (6.7) | 75 (36.2)  127 (31.7) | 123 (59.4)  246 (61.5) | 0.32 | 0.225  0.226 | 0.775  0.774 | 0.95 |
| **FH-HC** Absent  Present | 409  197 | 23 (5.6)  13 (6.6) | 138 (33.7)  63 (32.0) | 248 (60.4)  121 (61.4) | 0.84 | 0.225  0.226 | 0.775  0.774 | 0.97 |
| **FH-HG** Absent  Present | 594  12 | 32 (5.4)  4 (33.3) | 198 (33.3)  3 (25.0) | 364 (61.3)  5 (41.7) | 0.01 | 0.220  0.458 | 0.780  0.542 | 0.01 |
| **FH-DM** Absent  Present | 176  432 | 11 (6.2)  25 (5.8) | 58 (33.0)  144 (33.3) | 107 (60.8)  263 (60.9) | 0.98 | 0.227  0.224 | 0.773  0.776 | 0.92 |
| **FH-HD** Absent  Present | 405  203 | 23 (5.7)  13 (6.4) | 136 (33.6)  66 (32.5) | 246 (60.7)  124 (61.1) | 0.92 | 0.225  0.227 | 0.775  0.773 | 0.94 |

N = Number of sample, %= percentage of total, f=allele frequency

AP Arabian Peninsula Ancestry; APB Arabian Peninsula Bedouin Ancestry; HU Admix ethnicity; IR Persian Ancestry; BMI body mass index; TC total cholesterol; TG triglycerides; LDL-C low density lipoprotein-cholesterol; HDL-C high density lipoprotein-cholesterol; FM positive family history of HT hypertension, HC hypercholesterolemia, HG hypertriglyceridemia, DM diabetes mellitus 2, HD heart disease.

*The p-values were determined by chi-square test.

Table A.2.: Frequency distribution with regards to the mean and standard deviation (SE) of serum lipid levels using univariate Anova (UNIANOVA).

| **Variables** | **N** | **GENOTYPE** | | | | **ALLELE** | | |
| --- | --- | --- | --- | --- | --- | --- | --- | --- |
|  |  | DD | ID | II | p-value* | D | I | p-value* |
|  |  | Mean ± SE | Mean ± SE | Mean ± SE |  | Mean ± SE | Mean ± SE |  |
| Age | 789 | 31.70 ± 2.21 | 30.82 ± 0.86 | 31.78 ± 0.65 | 0.67 | 31.05 ± 0.75 | 31.57 ± 0.40 | 0.53 |
| BMI | 533 | 27.11 ± 1.08 | 27.29 ± 0.48 | 26.49 ± 0.35 | 0.39 | 27.24 ± 0.40 | 26.67 ± 0.22 | 0.21 |
| TC | 666 | 4.67 ± 0.15 | 4.69 ± 0.06 | 4.71 ± 0.05 | 0.90 | 4.68 ± 0.06 | 4.71 ± 0.03 | 0.65 |
| TG | 666 | 1.14 ± 0.13 | 0.95 ± 0.05 | 1.11 ± 0.04 | 0.04 | 1.00 ± 0.05 | 1.08 ± 0.03 | 0.15 |
| HDL-C | 629 | 1.01 ± 0.05 | 1.17 ± 0.02 | 1.11 ± 0.01 | 0.03 | 1.14 ± 0.02 | 1.12 ± 0.01 | 0.31 |
| LDL-C | 627 | 3.15 ± 0.14 | 3.10 ± 0.06 | 3.13 ± 0.04 | 0.89 | 3.11 ± 0.05 | 3.12 ± 0.03 | 0.83 |

*Two-tailed (t-test) p-value. A significant value of less than 5%**Table B: Distribution of the genotypic and allelic frequencies of the APOB 2488-XbaI C(X^-^)<T(X^+^) polymorphism (rs693) with serum lipid levels in the Kuwaiti population (n=795).**

B.1 Frequency distribution against all the variables analyzed in the study. The serum lipids were categorized based on the Kuwaiti population reference values (mmol/L) as indicated in the tables.

| **Variables** | **N** | **GENOTYPE** | | | | **ALLELE** | | |
| --- | --- | --- | --- | --- | --- | --- | --- | --- |
|  |  | X^+^X^-^ | X^+^X^+^ | X^-^X^-^ | p-value* | X^-^ | X^+^ | p-value* |
|  |  | N (%) | N (%) | N (%) |  | f | f |  |
| **Gender** Male  Female | 329  462 | 129 (39.2)  178 (38.5) | 20 (6.1)  37 (8.0) | 180 (54.7)  247 (53.5) | 0.58 | 0.743  0.727 | 0.257  0.273 | 0.48 |
| **TC** Normal (≤5.17)  High (>5.17) | 475  191 | 199 (41.9)  73 (38.2) | 37 (7.8)  14 (7.3) | 239 (50.3)  104 (54.4) | 0.63 | 0.713  0.736 | 0.287  0.264 | 0.40 |
| **TG** Normal (≤2.19)  High (>2.19) | 617  49 | 253 (41.0  19 (38.8) | 48 (7.8)  3 (6.1) | 316 (51.2)  27 (55.1) | 0.84 | 0.717  0.745 | 0.283  0.255 | 0.55 |
| **HDL-C** Normal (>1.3)  Abnormal (≤1.3) | 276  353 | 112 (40.6)  140 (39.7) | 21 (7.6)  28 (7.9) | 143 (51.8)  185 (52.4) | 0.97 | 0.721  0.722 | 0.279  0.278 | 0.96 |
| **LDL-C** Normal (≤3.2)  High (>3.2) | 389  238 | 152 (39.1)  98 (41.2) | 31 (8.0)  18 (7.6) | 206 (52.9)  122 (51.2) | 0.87 | 0.723  0.718 | 0.275  0.282 | 0.81 |
| **FH-HT** Absent  Present | 207  400 | 72 (34.8)  160 (40.0) | 13 (6.3)  29 (7.2) | 122 (58.9)  211 (52.7) | 0.35 | 0.763  0.727 | 0.237  0.273 | 0.18 |
| **FH-HC** Absent  Present | 409  197 | 153 (37.4)  79 (40.1) | 30 (7.3)  12 (6.1) | 226 (55.3)  106 (53.8) | 0.74 | 0.740  0.740 | 0.260  0.260 | 0.97 |
| **FH-HG** Absent  Present | 594  12 | 229 (38.6)  3 (25.0) | 39 (6.6)  3 (25.0) | 326 (54.9)  6 (50.0) | 0.12 | 0.742  0.625 | 0.258  0.375 | 0.22 |
| **FH-DM** Absent  Present | 176  432 | 61 (34.7)  171 (39.6) | 12 (7.4)  29 (6.7) | 102 (57.9)  232 (53.7) | 0.52 | 0.753  0.735 | 0.247  0.265 | 0.52 |
| **FH-HD** Absent (0)  Present (1) | 405  203 | 162 (40.0)  70 (34.5) | 29 (7.2)  13 (6.4) | 214 (52.8)  120 (59.1) | 0.34 | 0.728  0.763 | 0.272  0.237 | 0.10 |

N = Number of sample, %= percentage of total, f= frequency

AP Arabian Peninsula Ancestry; APB Arabian Peninsula Bedouin Ancestry; HU Admix ethnicity; IR Persian Ancestry; BMI body mass index; TC total cholesterol; TG triglycerides; LDL-C low density lipoprotein-cholesterol; HDL-C high density lipoprotein-cholesterol; FM positive family history of HT hypertension, HC hypercholesterolemia, HG hypertriglyceridemia, DM diabetes mellitus 2, HD heart disease.

*The p-values were determined by chi-square test.

Table B.2. Frequency distribution with regards to the mean and standard deviation (SE) of serum lipid levels using univariate Anova (UNIANOVA). A two-tailed (t-test) p-value of 0.05 was considered as statistical significance.

| **Parameters** | **N** | **GENOTYPE** | | | | **ALLELE** | | |
| --- | --- | --- | --- | --- | --- | --- | --- | --- |
|  |  | X^+^X^-^ | X^+^X^+^ | X^-^X^-^ | p-value* | X^-^ | X^+^ | p-value* |
|  |  | Mean ± SE | Mean ± SE | Mean ± SE |  | Mean ± SE | Mean ± SE |  |
| Age | 789 | 31.58 ± 0.81 | 30.82 ± 1.87 | 31.45 ± 0.69 | 0.93 | 31.49 ± 0.42 | 31.38 ± 0.69 | 0.89 |
| BMI | 533 | 27.03 ± 0.44 | 26.59 ± 1.02 | 26.65 ± 0.37 | 0.72 | 26.75 ± 0.23 | 26.91 ± 0.38 | 0.72 |
| TC | 666 | 4.72 ± 0.06 | 4.60 ± 0.14 | 4.70 ± 0.05 | 0.73 | 4.71 ± 0.03 | 4.69 ± 0.05 | 0.75 |
| TG | 666 | 1.08 ± 0.05 | 0.97 ± 0.11 | 1.06 ± 0.05 | 0.72 | 1.06 ± 0.03 | 1.05 ± 0.04 | 0.79 |
| HDL | 629 | 1.13 ± 0.02 | 1.13 ± 0.04 | 1.12 ± 0.02 | 0.89 | 1.12 ± 0.01 | 1.13 ± 0.02 | 0.67 |
| LDL | 627 | 3.12 ± 0.05 | 3.04 ± 0.12 | 3.13 ± 0.05 | 0.76 | 3.13 ± 0.03 | 3.10 ± 0.04 | 0.54 |

*Two-tailed (t-test) p-value.**Table C: Distribution of the genotypic and allelic frequencies of the APOB 3611-MspI G(M^+^)<A(M^-^) polymorphism (rs1801701) with serum lipid levels in the Kuwaiti population (n=795).**

C.1 Frequency distribution against all the variables analyzed in the study. The serum lipids were categorized based on the Kuwaiti population reference values (mmol/L) as indicated in the tables.

| **Variables** | **N** | **GENOTYPE** | | | | **ALLELE** | | |
| --- | --- | --- | --- | --- | --- | --- | --- | --- |
|  |  | M+M- | M+M+ | M-M- | p-value* | M- | M+ | p-value* |
|  |  | N (%) | N (%) | N (%) |  | f | f |  |
| **Gender** Male  Female | 329  462 | 41 (12.5)  57 (12.3) | 287 (87.2)  402 (87.0) | 1 (0.3)  3 (0.7) | 0.79 | 0.065  0.068 | 0.935  0.932 | 0.82 |
| **TC** Normal (≤5.17)  High (>5.17) | 475  191 | 65 (13.7)  18 (9.4) | 407 (85.7)  172 (90.0) | 3 (0.6)  1 (0.5) | 0.30 | 0.075  0.052 | 0.925  0.948 | 0.13 |
| **TG** Normal (≤2.19)  High (>2.19) | 617  49 | 82 (13.3)  1 (2.0) | 532 (86.2)  47 (95.9) | 3 (0.5)  1 (2.0) | 0.01 | 0.071  0.031 | 0.929  0.969 | 0.09 |
| **HDL-C** Normal (>1.3)  Abnormal (≤1.3) | 276  353 | 41 (14.9)  36 (10.2) | 234 (84.8)  314 (89.0) | 1 (0.4)  3 (0.8) | 0.16 | 0.078  0.059 | 0.922  0.941 | 0.20 |
| **LDL-C** Normal (≤3.2)  High (>3.2) | 389  238 | 49 (12.6)  28 (11.8) | 337 (86.6)  209 (87.8) | 3 (0.8)  1 (0.4) | 0.82 | 0.071  0.063 | 0.929  0.937 | 0.60 |
| **FH-HT** Absent  Present | 207  400 | 27 (13.0)  54 (13.5) | 178 (86.0)  346 (86.5) | 2 (1.0)  0 (0.0) | 0.12 | 0.075  0.068 | 0.925  0.932 | 0.64 |
| **FH-HC** Absent  Present | 409  197 | 45 (11.0)  35 (17.8) | 362 (88.5)  162 (82.3) | 2 (0.5)  0 (0.0) | 0.04 | 0.060  0.089 | 0.940  0.911 | 0.07 |
| **FH-HG** Absent  Present | 594  12 | 76 (12.8)  4 (33.3) | 516 (86.9)  8 (66.7) | 2 (0.3)  0 (0.0) | 0.19 | 0.067  0.166 | 0.933  0.833 | 0.10 |
| **FH-DM** Absent  Present | 176  432 | 17 (9.7)  64 (14.8) | 158 (89.8)  367 (85.0) | 1 (0.6)  1 (0.2) | 0.18 | 0.054  0.076 | 0.946  0.924 | 0.16 |
| **FH-HD** Absent  Present | 405  203 | 56 (13.8)  25 (12.3) | 348 (85.9)  177 (87.2) | 1 (0.2)  1 (0.5) | 0.78 | 0.072  0.066 | 0.928  0.934 | 0.74 |

N = Number of sample, %= percentage of total, f= frequency

AP Arabian Peninsula Ancestry; APB Arabian Peninsula Bedouin Ancestry; HU Admix ethnicity; IR Persian Ancestry; BMI body mass index; TC total cholesterol; TG triglycerides; LDL-C low density lipoprotein-cholesterol; HDL-C high density lipoprotein-cholesterol; FM positive family history of HT hypertension, HC hypercholesterolemia, HG hypertriglyceridemia, DM diabetes mellitus 2, HD heart disease.

*The p-values were determined by chi-square test.

Table C.2: Frequency distribution with regards to the mean and standard deviation (SE) of serum lipid levels using univariate Anova (UNIANOVA).

| **Variables** | **N** | **GENOTYPE (t-test)** | | | | **ALLELE (t-test)** | | |
| --- | --- | --- | --- | --- | --- | --- | --- | --- |
|  |  | M^+^M^-^ | M^+^M^+^ | M^-^M^-^ | p-value* | M^-^ | M^+^ | P-value* |
|  |  | Mean ± SE | Mean ± SE | Mean ± SE |  | Mean ± SE | Mean ± SE |  |
| Age | 789 | 28.79 ± 1.42 | 31.83 ± 0.54 | 33.00 ± 7.07 | 0.14 | 29.11 ± 1.37 | 31.63 ± 0.36 | 0.08 |
| BMI | 533 | 25.70 ± 0.76 | 26.93 ± 0.29 | 34.28 ± 4.50 | 0.08 | 26.17 ± 0.75 | 26.84 ± 0.20 | 0.39 |
| TC | 666 | 4.66 ± 4.45 | 4.71 ± 0.04 | 4.27 ± 0.49 | 0.60 | 4.63 ± 0.10 | 4.71 ± 0.03 | 0.42 |
| TG | 666 | 0.88 ± 0.09 | 1.08 ± 0.03 | 1.41 ± 0.41 | 0.07 | 0.92 ± 0.09 | 1.07 ± 0.02 | 0.11 |
| HDL-C | 629 | 1.16 ± 0.04 | 1.12 ± 0.01 | 1.07 ± 0.16 | 0.56 | 1.15 ± 0.03 | 1.12 ± 0.01 | 0.41 |
| LDL-C | 627 | 3.09 ± 0.09 | 3.13 ± 0.03 | 2.53 ± 0.41 | 0.35 | 3.04 ± 0.09 | 3.12 ± 0.02 | 0.361 |

*Two-tailed (t-test) p-value. A significant value of less than 5%; A trend for possible significance **Table D: Distribution of the genotypic and allelic frequencies of the APOB 4154-EcoRI G(E^+^)<A(E^-^) polymorphism (rs1042031) with serum lipid levels in the Kuwaiti population (n=795).**

D.1 Frequency distribution against all the variables analyzed in the study. The serum lipids were categorized based on the Kuwaiti population reference values (mmol/L) as indicated in the tables.

| **Variables** | **N** | **GENOTYPE** | | | | **ALLELE** | | |
| --- | --- | --- | --- | --- | --- | --- | --- | --- |
|  |  | E^+^E^-^ | E^+^E^+^ | E^-^E^-^ | p-value* | E^-^ | E^+^ | p-value* |
|  |  | N (%) | N (%) | N (%) |  | f | f |  |
| **Gender** Male  Female | 329  462 | 57 (17.3)  85 (18.4) | 265 (80.5)  371 (80.3) | 7 (2.1)  6 (1.3) | 0.63 | 0.108  0.105 | 0.892  0.895 | 0.85 |
| **TC** Normal (≤5.17)  High (>5.17) | 475  191 | 83 (17.5)  35 (18.3) | 383 (80.6)  154 (80.6) | 9 (1.9)  2 (1.0) | 0.70 | 0.106  0.102 | 0.894  0.898 | 0.82 |
| **TG** Normal (≤2.19)  High (>2.19) | 617  49 | 113 (18.3)  5 (10.2) | 493 (79.9)  44 (80.6) | 11 (1.8)  0 (0.0) | 0.12 | 0.109  0.051 | 0.891  0.949 | 0.05 |
| **HDL-C** Normal (>1.3)  Abnormal (≤1.3) | 276  353 | 48 (17.4)  63 (17.8) | 223 (80.8)  285 (80.7) | 5 (1.8)  5 (1.4) | 0.92 | 0.105  0.103 | 0.895  0.897 | 0.92 |
| **LDL-C** Normal (≤3.2)  High (>3.2) | 389  238 | 63 (16.2)  48 (20.2) | 319 (82.0)  187 (78.6) | 7 (1.8)  3 (1.3) | 0.41 | 0.099  0.113 | 0.901  0.887 | 0.42 |
| **FH-HT** Absent  Present | 207  400 | 43 (20.8)  73 (18.2) | 159 (76.8)  320 (80.0) | 5 (2.4)  7 (1.8) | 0.63 | 0.128  0.109 | 0.872  0.891 | 0.32 |
| **FH-HC** Absent  Present | 409  197 | 82 (20.0)  33 (16.8) | 321 (78.5)  158 (80.2) | 6 (1.5)  6 (3.0) | 0.30 | 0.115  0.114 | 0.885  0.886 | 0.97 |
| **FH-HG** Absent  Present | 594  12 | 114 (19.2)  1 (8.3) | 468 (78.8)  11 (91.7) | 12 (2.0)  0 (0.0 | 0.44 | 0.116  0.042 | 0.884  0.958 | 0.20 |
| **FH-DM** Absent  Present | 176  432 | 28 (15.9)  88 (20.4) | 144 (81.8)  336 (77.8) | 4 (2.3)  8 (1.8) | 0.42 | 0.102  0.120 | 0.898  0.880 | 0.37 |
| **FH-HD** Absent  Present | 405  203 | 76 (18.8)  40 (19.7) | 321 (79.3)  159 (78.3) | 8 (1.9)  4 (2.0) | 0.96 | 0.114  0.118 | 0.886  0.882 | 0.81 |

N = Number of sample, %= percentage of total, f= frequency

AP Arabian Peninsula Ancestry; APB Arabian Peninsula Bedouin Ancestry; HU Admix ethnicity; IR Persian Ancestry; BMI body mass index; TC total cholesterol; TG triglycerides; LDL-C low density lipoprotein-cholesterol; HDL-C high density lipoprotein-cholesterol; FM positive family history of HT hypertension, HC hypercholesterolemia, HG hypertriglyceridemia, DM diabetes mellitus 2, HD heart disease.

*The p-values were determined by chi-square test.

Table D.2: Frequency distribution with regards to the mean and standard deviation (SE) of serum lipid levels using univariate Anova (UNIANOVA).

| **Variables** | **N** | **GENOTYPE** | | | | **ALLELE** | | |
| --- | --- | --- | --- | --- | --- | --- | --- | --- |
|  |  | E^+^E^-^ | E^+^E^+^ | E^-^E^-^ | P value* | E^-^ | E^+^ | P value* |
|  |  | Mean ± SE | Mean ± SE | Mean ± SE |  | Mean ± SE | Mean ± SE |  |
| Age | 789 | 32.10 ± 1.19 | 31.32 ± 0.56 | 31.15 ± 3.93 | 0.84 | 31.95 ± 1.09 | 31.40 ± 0.38 | 0.63 |
| BMI | 533 | 28.03 ± 0.63 | 26.51 ± 0.31 | 26.35 ± 2.11 | 0.10 | 27.78 ± 0.58 | 26.68 ± 0.21 | 0.08 |
| TC | 666 | 4.74 ± 0.09 | 4.700 ± 0.04 | 4.52 ± 0.29 | 0.28 | 4.71 ± 0.08 | 4.70 ± 0.03 | 0.96 |
| TG | 666 | 1.06 ± 0.08 | 1.06 ± 0.04 | 1.03 ± 0.25 | 0.99 | 1.05 ± 0.07 | 1.06 ± 0.02 | 0.92 |
| HDL-C | 629 | 1.11 ± 0.03 | 1.13 ± 0.01 | 1.10 ± 0.10 | 0.85 | 1.11 ± 0.03 | 1.13 ± 0.01 | 0.56 |
| LDL-C | 627 | 3.18 ± 0.08 | 3.12 ± 0.04 | 3.07 ± 0.26 | 0.73 | 3.16 ± 0.07 | 3.12 ± 0.02 | 0.56 |

*Two-tailed (t-test) p-value. A significant value of less than 5%; A trend for possible significance

**Table E: Distribution of the genotypic and allelic frequencies of the APOB VNTR medium(M)/long(L) polymorphism with serum lipid levels in the Kuwaiti population (n=795).**

E.1 Frequency distribution against all the variables analyzed in the study. The serum lipids were categorized based on the Kuwaiti population reference values (mmol/L) as indicated in the tables.

| **Variables** | **N** | **GENOTYPE** | | | | **ALLELE** | | |
| --- | --- | --- | --- | --- | --- | --- | --- | --- |
|  |  | L/L | M/L | M/M | p-value* | L | M | p-value* |
|  |  | N (%) | N (%) | N (%) |  | f | f |  |
| **Gender** Male  Female | 329  462 | 9 (2.7)  15 (3.2) | 52 (15.8)  64 (13.8) | 268 (81.5)  383 (82.9) | 0.70 | 0.106  0.102 | 0.894  0.898 | 0.77 |
| **TC** Normal (≤5.17)  High (>5.17) | 475  191 | 16 (3.4)  4 (2.1) | 68 (14.3)  30 (15.7) | 391 (82.3)  157 (82.2) | 0.62 | 0.105  0.099 | 0.895  0.901 | 0.75 |
| **TG** Normal (≤2.19)  High (>2.19) | 617  49 | 20 (3.2)  0 (0.0) | 92 (14.9)  6 (12.2) | 505 (81.9)  43 (87.8) | 0.18 | 0.107  0.061 | 0.893  0.939 | 0.13 |
| **HDL-C** Normal (>1.3)  Abnormal (≤1.3) | 276  353 | 10 (3.6)  8 (2.3) | 40 (14.5)  52 (14.7) | 226 (81.9)  293 (83.0) | 0.60 | 0.109  0.096 | 0.891  0.904 | 0.47 |
| **LDL-C** Normal (≤3.2)  High (>3.2) | 389  238 | 12 (3.1)  6 (2.5) | 56 (14.4)  35 (14.7) | 321 (82.5)  197 (82.8) | 0.92 | 0.103  0.099 | 0.897  0.901 | 0.82 |
| **FH-HT** Absent  Present | 207  400 | 7 (3.4)  14 (3.5) | 34 (16.4)  44 (11.0) | 166 (80.2)  342 (85.5) | 0.18 | 0.116  0.090 | 0.884  0.910 | 0.16 |
| **FH-HC** Absent  Present | 409  197 | 14 (3.4)  7 (3.5) | 56 (13.7)  22 (11.2) | 339 (82.9)  168 (85.3) | 0.68 | 0.103  0.091 | 0.897  0.909 | 0.53 |
| **FH-HG** Absent  Present | 594  12 | 21 (3.5)  0 (0.0 | 77 (13.0)  1 (8.3) | 496 (83.5)  11 (91.7) | 0.56 | 0.100  0.042 | 0.900  0.958 | 0.29 |
| **FH-DM** Absent  Present | 176  432 | 6 (3.4)  15 (3.5) | 19 (10.8)  59 (13.7) | 151 (85.8)  358 (82.9) | 0.62 | 0.088  0.103 | 0.912  0.897 | 0.42 |
| **FH-HD** Absent  Present | 405  203 | 17 (4.2)  4 (2.0) | 48 (11.8)  30 (14.8) | 340 (84.0)  169 (83.2) | 0.22 | 0.101  0.094 | 0.899  0.906 | 0.67 |

N = Number of sample, %= percentage of total, f= frequency

AP Arabian Peninsula Ancestry; APB Arabian Peninsula Bedouin Ancestry; HU Admix ethnicity; IR Persian Ancestry; BMI body mass index; TC total cholesterol; TG triglycerides; LDL-C low density lipoprotein-cholesterol; HDL-C high density lipoprotein-cholesterol; FM positive family history of HT hypertension, HC hypercholesterolemia, HG hypertriglyceridemia, DM diabetes mellitus 2, HD heart disease.

*The p-values were determined by chi-square test.

Table E.2 Frequency distribution with regards to the mean and standard deviation (SE) of serum lipid levels using univariate Anova (UNIANOVA).

| **Variables** | **N** | **GENOTYPE** | | | | **ALLELE** | | |
| --- | --- | --- | --- | --- | --- | --- | --- | --- |
|  |  | L/L | M/L | M/M | p-value* | L | M | p-value* |
|  |  | Mean ± SE | Mean ± SE | Mean ± SE |  | Mean ± SE | Mean ± SE |  |
| Age | 789 | 33.21 ± 2.89 | 29.96 ± 1.31 | 31.66 ± 0.56 | 0.41 | 30.91 ± 1.10 | 31.52 ± 0.37 | 0.60 |
| BMI | 533 | 26.23 ± 1.46 | 27.50 ± 0.79 | 26.72 ± 0.30 | 0.61 | 27.03 ± 0.63 | 26.78 ± 0.21 | 0.71 |
| TC | 666 | 4.52 ± 0.22 | 4.60 ± 1.00 | 4.73 ± 0.04 | 0.34 | 4.58 ± 0.08 | 4.72 ± 0.03 | 0.11 |
| TG | 666 | 0.94 ± 0.19 | 1.02 ± 0.08 | 1.07 ± 0.04 | 0.70 | 1.00 ± 0.07 | 1.07 ± 0.02 | 0.35 |
| HDL-C | 629 | 1.16 ± 0.07 | 1.09 ± 0.03 | 1.13 ± 0.01 | 0.46 | 1.11 ± 0.03 | 1.13 ± 0.01 | 0.55 |
| LDL-C | 627 | 3.00 ± 0.19 | 3.12 ± 0.09 | 3.13 ± 0.04 | 0.83 | 3.08 ± 0.07 | 3.13 ± 0.02 | 0.60 |

*Two-tailed (t-test) p-value.
